# Experimental parasite community perturbation reveals associations between Sin Nombre virus and gastrointestinal nematodes in a rodent reservoir host

**DOI:** 10.1098/rsbl.2020.0604

**Published:** 2020-12-23

**Authors:** Amy R. Sweeny, Courtney A. Thomason, Edwin A. Carbajal, Christina B. Hansen, Andrea L. Graham, Amy B. Pedersen

**Affiliations:** 1 Institute of Evolutionary Biology and Centre of Infection, School of Biological Sciences, Kings Buildings, Ashworth Laboratories, Charlotte Auerbach Road, Edinburgh, UK; 2 Department of Biological Sciences, Texas Tech University, Lubbock, TX, USA; 3 Department of Biological Sciences, Virginia Polytechnic Institute and State University, Blacksburg, VA, USA; 4 Department of Microbiology, Icahn School of Medicine at Mount Sinai, New York, NY, USA; 5 Department of Ecology and Evolutionary Biology, Princeton University, Princeton, NJ, USA

**Keywords:** Sin Nombre virus, gastrointestinal nematodes, disease control, reservoir host, co-infection

## Abstract

Individuals are often co-infected with several parasite species, yet measuring within-host interactions remains difficult in the wild. Consequently, the impacts of such interactions on host fitness and epidemiology are often unknown. We used anthelmintic drugs to experimentally reduce nematode infection and measured the effects on both nematodes and the important zoonosis Sin Nombre virus (SNV) in its primary reservoir (*Peromyscus* spp.). Treatment significantly reduced nematode infection, but increased SNV seroprevalence. Furthermore, mice that were co-infected with both nematodes and SNV were in better condition and survived up to four times longer than uninfected or singly infected mice. These results highlight the importance of investigating multiple parasites for understanding interindividual variation and epidemiological dynamics in reservoir populations with zoonotic transmission potential.

## 1. Introduction

Co-infection with both microparasites and macroparasites is common in the wild [1,2]. Interactions among parasites co-habiting a host can occur through multiple mechanisms, including bottom-up (e.g. resource competition) or top-down (e.g. immune-mediated) processes [3–5]. These interactions can alter both host and parasite fitness [1,2,6,7], e.g. increasing parasite burdens for a co-infecting species [8,9], worsening disease pathology [6], altering transmission rates [10] and ultimately influencing the efficacy of disease control strategies [11].

Disease ecologists commonly assess the consequences of infection in a wild host by removing a target parasite group using drug treatments [12], but monitoring the non-target parasite community response is rarer. Some studies have used perturbation experiments to determine the strength and direction of within-host parasite interactions by measuring the response of non-targeted parasite species after treatment [8,9,13]. In African buffalo (*Syncerus caffer*), animals treated to remove nematodes were nine times more likely to survive co-infection with the bacterium *Mycobacterium tuberculosis* [13]. By contrast, removal of nematodes in wild rodents has been shown to increase coccidian microparasite infection, possibly through competitive release [8,9]. These studies show that ignoring the broader parasite community may crucially underestimate the occurrence and importance of within-host interactions. For interactions between parasites with zoonotic potential or that severely impair the immune system—as with HIV and the re-emergence of drug-resistant tuberculosis—the public health implications can be severe [14]. Mechanistic insights from systems where experimental approaches are possible will be key for the understanding of the role of co-infection in natural populations [15,16].

Small mammals disproportionately serve as reservoir species for zoonotic diseases [17–20] and are ideal, tractable systems for experimental studies. Hantavirus pulmonary syndrome (HPS) is a zoonotic disease caused by Sin Nombre virus (SNV), endemic in deer mice (*Peromyscus maniculatus*) and white-footed mice (*Peromyscus leucopus*) [21,22]. Hantavirus infection can reduce wild rodent fitness [22,23] and often co-occurs with other endemic parasites [24,25]. Here, we experimentally perturbed the taxonomically diverse parasite communities of deer and white-footed mice*,* the primary wild reservoirs of SNV [21]. Nematodes represent keystone parasites in within-host communities because they can interact with other parasites through the host immune system or through direct competition for resources in the gastrointestinal (GI) tract [4,26]. Previous work in this system found that GI nematode infections were common and interacted with other co-infecting GI- and ectoparasites [27,28]. We used anthelmintic treatment to remove nematodes and monitored downstream effects on SNV infection and host fitness. We show that removal of nematode infections increases the subsequent probability of SNV seroconversion and that co-infection with nematodes and SNV conveys condition and survival benefits within this population.

## 2. Methods

Field experiments were conducted at the Mountain Lake Biological Station in southwest Virginia, where populations of deer and white-footed mice have been monitored for decades [29], and the parasite community is well-characterized [9,27]. Live-trapping took place from May/June to August in two temporal replicates (2010 and 2011) on three spatial replicates of two, 0.5 ha grids each (8 × 8 trap arrays; 10 m spacing). Each grid set was trapped for three consecutive nights every two weeks. In each temporal replicate, randomized anthelmintic treatment was administered at first capture and repeated fortnightly with a weight-adjusted oral dose of ivermectin (5 mg kg^−1^; Eqvalan, Merial, USA) or control (5% sucrose solution). See the electronic supplementary material for additional details.

At first capture, individuals were ear-tagged and their species identified using morphological characteristics [30]. At each capture, morphometric data (age, sex, weight, reproductive condition) were recorded. Faecal and blood samples were also collected fortnightly. The presence/absence and number of eggs per gram of faeces (a common proxy of infection intensity; EPG) for nematode species were quantified using a salt flotation technique [31]. Nematode species were aggregated for analysis because drug treatment is at the group (nematode) level. Blood samples were screened for SNV antibodies using standard enzyme-linked immunosorbent assay (ELISA) protocols and reagents from the U.S. Centers for Disease Control and Prevention [32,33]. ELISA results were used to assign infection status based on seropositivity (presence/absence: threshold of 3 s.d. greater than negative control) and for positive samples the adjusted optical density (OD) relative to a negative control (CDC no. 703226) was used to estimate antibody concentration for statistical analyses. Additional details are given in the electronic supplementary material.

All statistical analyses were conducted in R v. 3.6.0 [34]. We first investigated factors driving natural SNV and nematode infections prior to experimental perturbations by fitting generalized linear mixed-effects models (GLMMs) using the package ‘glmmTMB’ to SNV (both presence/absence and antibody concentration) or nematodes (both presence/absence and intensity (EPG)) for all first capture events. Models were fitted with binomial (logit link; SNV and nematode presence/absence), Gaussian (SNV OD-positive only) or negative binomial (log link; nematode intensity, infected only) error distributions. We included the following fixed effects: year (factor: 2010/2011), Julian date of capture (continuous, scaled to mean = 0/s.d. = 1), sex (factor: male/female), age (factor: sub-adult/adult), species (factor: *P. leucopus*/*P. maniculatus*) and body weight (continuous, grams). Nematode presence (factor: 0/1) was included in SNV models to test for influence of nematode presence prior to treatment.

We then tested the relationship between SNV and GI nematodes by fitting GLMMs to the same response variables detailed above, using data from all captures and including additional fixed effects of treatment (factor: ivermectin treated/control), nematode infection status (factor: present/absent) and an interaction of treatment with timepoint (factor: pre-/post-treatment). Additional model details are given in the electronic supplementary material.

Finally, we investigated the effects of drug treatment and SNV–nematode co-infection on host body weight, as a proxy of the condition, and recapture duration (number of days known alive) as a proxy for survival using a GLMM with Gaussian and negative binomial error distributions, respectively. The following fixed effects were included for both models: year, sex, age, species, treatment (all as described above), and infection status (factor, 4-level: none, SNV only, nematode only, co-infected). Body condition models included additional effects of reproductive status and a random effect of individual ID, while survival models included additional fixed effects of weight and trap session across both years (continuous, 1–11) to account for skewed observation times. Grid (6-level factor) was included as a random effect in all models to account for variation across spatial replicates.

## 3. Results

Four hundred and nine individuals were captured in total (*n*_2010_ = 186; *n*_2011_ = 223, table 1). Prior to anthelmintic treatment, SNV prevalence was 10.3% and GI nematode prevalence was 28.4%. Mouse sex, body weight and capture date were the primary determinants of both SNV infection probability and antibody concentration before treatment (table 2). Males (sex, male: infection probability—*β* = 0.89, s.e. = 0.39, *p* = 0.022; titre—*β* = 0.11, s.e. = 0.06, *p* = 0.09) and larger mice (weight (g): infection probability—*β* = 0.11, s.e. = 0.05, *p* = 0.015; titre—*β* = 0.02, s.e. = 0.01, *p* = 0.048) were more likely to be infected with SNV, while infection probability declined later in the summer (Julian date (scaled): infection probability—*β* = −0.51, s.e. = 0.21, *p* = 0.013; titre—*β* = −0.12, s.e. = 0.03, *p* < 0.001). There were no significant predictors of nematode infection probability at first capture (table 2), and only time of season was a significant predictor of nematode infection intensity (Julian date (scaled): *β* = 0.90, s.e. = 0.26, *p* < 0.001), where EPG increased throughout the summer.

**Table 1.** Field experiment population characteristics.

co-infection status			first capture, no. individuals				subsequent captures, no. captures (no. individuals)			
			none	nem. only	SNV only	co-infected	none	nem. only	SNV only	co-infected
host factors	males	adult	70	39	12	8	37 (29)	27 (20)	3 (2)	4 (2)
		sub-adult	60	19	6	3	12 (10)	6 (5)	0	0
	females	adult	51	37	6	4	36 (27)	29 (17)	5 (2)	6 (1)
		sub-adult	62	25	3	4	9 (6)	4 (4)	0	2 (2)
total no. individuals			243	120	27	19	72	46	4	5

**Table 2.** Model output for nematode and SNV dynamics at first capture and all captures of the experiment. Bold type indicates significance at the *p* < 0.05 threshold. Values in parentheses indicate (lower, upper) 95% CI.

	variable	nematodes				SNV			
		first capture		all captures		first capture		all captures	
		estimate	*p*	estimate	*p*	estimate	*p*	estimate	*p*
infection probability	ivermectin, treated : timepoint, post-treatment			−**2.17** **(**−**3.36**, **−0.98)**	**<0**.**001**			**1.61** (**0.16, 3.05)**	**0**.**029**
	ivermectin, treated			0.10 (−0.42, 0.63)	0.698			−0.19 (−1.04, 0.66)	0.659
	timepoint, post-treatment			0.49 (−0.06, 1.04)	0.084			−0.18 (−1.36, 1.00)	0.766
	year, 2011	−0.07 (−0.54, 0.39)	0.762	−0.06 (−0.48, 0.35)	0.758	−0.56 (−1.29, 0.17)	0.133	−0.51 (−1.17, 0.15)	0.127
	Julian date, scaled	−0.07 (−0.32, 0.18)	0.595	−0.14 (−0.37, 0.09)	0.225	−**0.51** (−**0.92**, −**0.11)**	**0**.**013**	−**0.46** (−**0.83, −0.08)**	**0**.**017**
	sex, male	−0.18 (−0.63, 0.28)	0.446	−0.12 (−0.50, 0.27)	0.547	**0.89** (**0.13, 1.65)**	**0**.**022**	0.44 (−0.20, 1.08)	0.180
	age, sub-adult	0.23 (−0.83, 0.37)	0.458	0.05 (−0.57, 0.47)	0.858	−0.21 (−0.78, 1.19)	0.683	−0.63 (−0.24, 1.5)	0.158
	species, *P. maniculatus*	0.35 (−0.29, 0.98)	0.280	0.38 (−0.19, 0.94)	0.191	−0.63 (−1.70, 0.45)	0.253	−0.27 (−1.18, 0.64)	0.564
	weight (g)	0.04 (−0.02, 0.10)	0.169	**0.06** (**0.01, 0.11)**	**0**.**018**	**0.11** (**0.02, 0.20)**	**0**.**015**	**0.15** (**0.08, 0.23)**	**<0**.**001**
	nematodes, present					0.18 (−0.56, 0.92)	0.636		
	intercept	−**1.53** (−**2.92,** −**0.14)**	**0**.**031**	−**2.07** (−**3.28,** −**0.85)**	**0**.**001**	−**5.12** (−**7.54,** −**2.70)**	**<0**.**001**	−**5.81** (−**7.90,** −**3.71)**	**<0**.**001**
infection intensity (nematodes) or antibody response (SNV)	ivermectin, treated : timepoint, post-treatment			−**5.21** (−**7.00,** −**3.43)**	**<0**.**001**			0.22 (−0.02, 0.47)	0.076
	ivermectin, treated			0.05 (−0.86, 0.95)	0.921			−0.01 (−0.16, 0.14)	0.944
	timepoint, post-treatment			0.10 (−0.91, 1.12)	0.841			0.00 (−0.17, 0.17)	0.968
	year, 2011	−0.39 (−0.89, 0.12)	0.135	−0.58 (−1.37, 0.21)	0.151	0.07 (−0.05, 0.20)	0.248	0.04 (−0.07, 0.16)	0.462
	Julian date, scaled	**0.90** (**0.62**, **1.17)**	**<0**.**001**	**0.82** (**0.36**, **1.29)**	**0**.**001**	−**0.12** (−**0.19**, −**0.06)**	**<0**.**001**	−**0.10** (−**0.16,** −**0.04)**	**0**.**001**
	sex, male	−0.48 (−1.05, 0.09)	0.096	−0.29 (−1.07, 0.50)	0.473	0.11 (−0.02, 0.23)	0.090	−0.01 (−0.12, 0.10)	0.847
	age, sub-adult	0.05 (−0.76, 0.60)	0.895	−0.07 (−1.05, 0.91)	0.889	0.11 (−0.29, 0.06)	0.204	−**0.15** **(0.13, 0.30)**	**0**.**038**
	species, *P. maniculatus*	0.21 (−0.49, 0.90)	0.563	0.96 (−0.18, 2.11)	0.100	0.08 (−0.15, 0.31)	0.491	0.19 (−0.01, 0.39)	0.059
	weight (g)	0.04 (−0.03, 0.11)	0.285	0.07 (−0.04, 0.18)	0.221	**0.02** (**0.00, 0.03)**	**0**.**048**	**0.02** (**0.01, 0.04)**	**0**.**001**
	nematodes, present					0.05 (−0.09, 0.19)	0.525		
	intercept	**4.82** (**3.06, 6.58)**	**<0**.**001**	**2.81** (**0.11, 5.52)**	**0**.**041**	−0.13 (−0.51, 0.25)	0.502	−0.17 (−0.48, 0.14)	0.277

**Figure 1.**
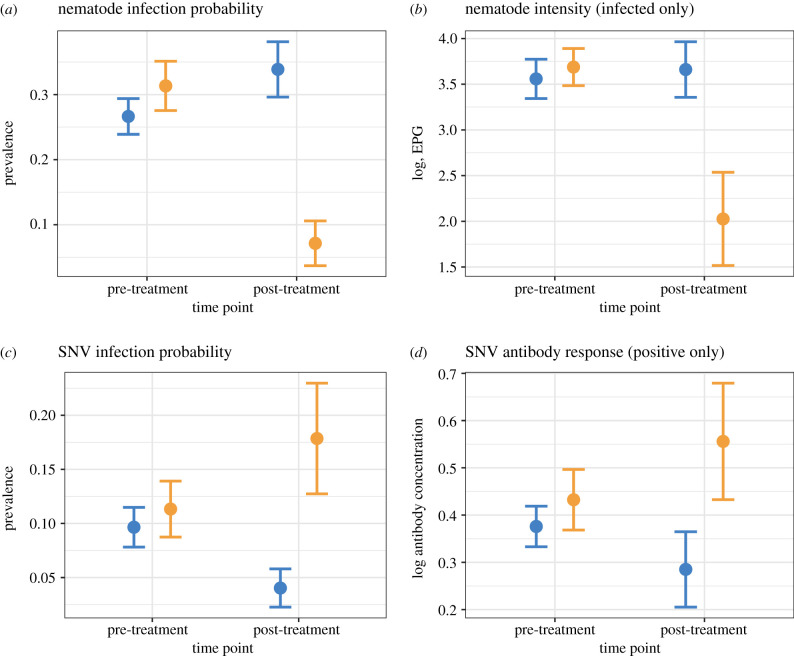
Anthelmintic treatment impacts nematode (*a,b*) and SNV (*c,d*) infection dynamics. Plots represent raw data for pre- or post-treatment groups. Treated mice (orange) had lower nematode infection probability (*a*) and intensity (*b*), but higher SNV infection probability (*c*) and antibody response (*d*). Points represent mean ± s.e.

Anthelmintic treatment reduced nematode infection probability (77.0% reduction) and intensity (89.8% reduction) (ivermectin, treated : timepoint, post-treatment: probability—*β* = −2.17, s.e. = 0.61, *p* < 0.001; intensity—*β* = −5.21, s.e. = 0.91, *p* < 0.001) (figure 1*a,b*). By contrast, SNV infection probability increased following anthelmintic treatment (54.5% increase, probability—*β* = 1.61, s.e. = 0.74, *p* = 0.029). SNV antibody levels were also greater following treatment; however, this was not significant (table 2). Nematode infection probability was also associated with body weight, where larger mice were more likely to be infected (table 2). As in pre-treatment models, capture date was a predictor of nematode infection intensity, and body weight and capture date were significant predictors of SNV infection probability and antibody concentration (table 2). Finally, sub-adult mice had higher SNV antibody response compared with adult mice (table 2).

**Figure 2.**
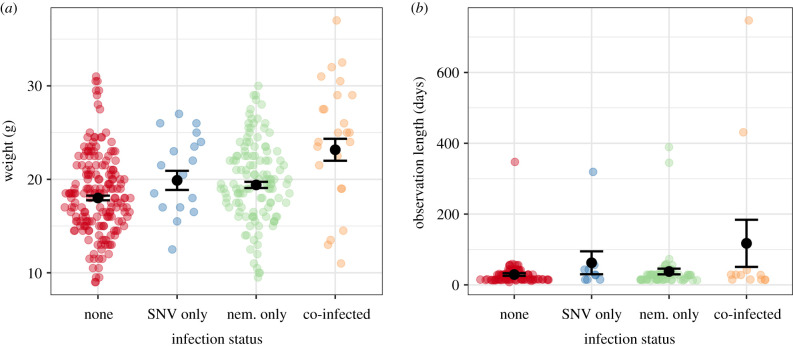
Co-infection effects on (*a*) host condition (weight (g)) and (*b*) host survival (days observed). Sina plots represent raw data distributions for infection groups: never infected, infected with either SNV or nematodes, or co-infected. Black point and error bars represent mean ± s.e.

We found positive effects of co-infection with SNV and nematodes on both host body weight as a proxy of host condition (weight (g): *β* = 3.37, s.e. = 0.89, *p* < 0.001) and recapture duration as a proxy for survival (observation length (days): *β* = 1.62, s.e. = 0.29, *p* < 0.001), where co-infected individuals on average were 3 g (20%) heavier and observed for four times longer than singly-infected individuals (figure 2). Weight variation with age was accounted for by including host age as a fixed effect (age class, adult: *β* = 5.04, s.e. = 0.35, *p* < 0.001). We found additional effects of sex (sex, male: *β* = −1.11, s.e. = 0.34, *p* = 0.001) and reproductive status (reproductive status, active: *β* = 1.62, s.e. = 0.31, *p* < 0.001) on body weight. In survival models, time to first capture was accounted for by including trap session as a fixed effect, but this was not a significant predictor of recapture duration (table S1).

## 4. Discussion

Efforts to understand the risk of emerging infectious disease from wildlife reservoirs commonly focus on anthropogenic or environmental factors that influence contact at the human–wildlife interface [35–37], while interindividual variation in susceptibility and transmission potential within reservoir hosts remains under-studied [38,39]. Within-host interactions among parasites can shape infection risk and fundamentally change pathogen virulence and transmission potential [15], but the influence of co-infection on zoonotic potential is still poorly understood. Here, we show that the loss of important nematode parasites drove increased prevalence of a zoonotic virus, demonstrating that co-infecting parasites could be an important mediating factor in transmission among reservoir species. This finding supports the idea that parasite diversity loss could result in increased zoonotic outbreaks [40] and that parasite conservation effects may be a valuable strategy in zoonotic disease control [41].

Mice that were co-infected with SNV and GI nematodes had higher body weight and were observed for longer than uninfected or singly infected individuals, which could alter disease dynamics by modifying infected individuals' transmission potential. For example, anthelminthic treatment in African buffalo (*S. caffer*) decreased mortality from bovine tuberculosis (BTB), resulting in an eightfold increase in the BTB reproductive number within the population [13]. Although hantaviruses are not considered to cause much pathology in rodents, evidence from *Peromyscus* spp. suggests that they can result in some associated mortality [22,23]. The enhanced condition and lifespan observed here may represent an unexpected benefit of nematode co-infection for wild rodents infected with SNV. There are some limitations of body weight as a proxy for body condition and observed time alive for survival. The condition was not scaled to length as body length was not available for each time point; however, we account for all common factors influencing host size in the model. For survival, we cannot rule out confounding effects such as dispersal or interindividual variation in trapping likelihood. Furthermore, a small number of mice (*N* = 10) survived for multiple years in this study and contributed disproportionately to observation length within the co-infected group. However, previous work suggests that SNV does not influence dispersal in deer mice [42] and no other host factors (e.g. sex, age, weight) impacted recapture duration in our study. We suggest that controlled experiments will be needed to explicitly test whether and how SNV–nematode co-infection prolongs survival.

Our observations imply that reducing the nematode burden creates beneficial conditions for SNV infection, potentially by altering the within-host immune environment. The immune response to nematodes is typically dominated by a combination of T-helper cell 2 (Th2) and T-helper cell regulatory (Treg) immune responses [43]. These responses include a suite of Th2-related cytokines, which are important mediators of inflammatory responses of the T helper cell 1 (Th1) arm of the immune system [44], and are better suited to minimize damage to the host rather than directly clear parasites, resulting in chronic infections [45]. Hantaviruses are likewise chronic in rodents and use a distinct mechanism to achieve immune evasion to persist and replicate in the absence of overt disease, whereby the virus may directly mediate suppression of Th1 responses via structural and non-structural proteins [46]. Protective antibody responses develop two weeks post hantavirus infection and can remain detectable throughout a rodent's life [46]. It is, therefore, possible that our results represent a reversal of nematode-induced immunosuppression following anthelmintic treatment. Given the infection status of SNV was determined by ELISA assays, it is also possible that these results reveal an increased detection probability of SNV due to the higher magnitude of response in the absence of nematode infection. However, given the detection of seroconversion is rare and only a small number of mice (4%) were detected as seroconverting in this population, we cannot definitively ascribe this mechanism. Alternatively, if chronic nematode infection imposes energetic costs, removing these parasites could result in greater host movement and sociality, driving greater SNV exposure [47,48]. Regardless of underlying mechanisms, these observed nematode–SNV interactions confirm that distantly related parasites can be mechanistically linked, and studies that do not consider co-infection may be missing an important source of variation in disease ecology [3,15,49].

## Supplementary Material

SNV-nematode associations in the wild Electronic Supplementary Material

## Data accessibility

Data for this manuscript are available on the Dryad Digital Repository: https://dx.doi.org/10.5061/dryad.6wwpzgmx2 [50].
