# Supplementary material for: Experimental parasite community perturbation reveals associations between Sin Nombre virus and gastrointestinal nematodes in a rodent reservoir host
Source: Biol Lett. 2020 Dec 23;16(12):20200604. doi: 10.1098/rsbl.2020.0604 (PMC7775983; doi:10.1098/rsbl.2020.0604)
Supplement: SNV-nematode associations in the wild Electronic Supplementary Material [file rsbl20200604supp1.pdf]

Experimental parasite community perturbation reveals associations between Sin Nombre virus and gastrointestinal nematodes in a rodent reservoir host

Amy R. Sweeny*, Courtney A. Thomason*, Edwin A. Carbajal, Christina B. Hansen, Andrea L. Graham, Amy B. Pedersen

Electronic Supplementary Material

Field Experiment & Data Collection

Mountain Lake Biological Station is located in an oak-maple forest in the Appalachian Mountains of southwestern Virginia (37°10'N, 80°20'W, 1200 m). Trapping was carried out in the summer months to correspond with the most active time of year for *Peromyscus spp.* Populations; beginning shortly after their first reproductive bout. Trapping nights consisted of baiting Sherman live traps (3" × 3.5" × 9", H. B. Sherman Traps, Tallahassee, FL) with crimped oats and cotton bedding on cool (<55°F) nights and checking for captures early the next morning (Figure S1). Animal processing and sampling was carried out in accordance with the Texas Tech University IACUC guidelines under protocol number 09013-4 and the Virginia Department of Game and Inland Fisheries small mammal trapping permit # 041919.

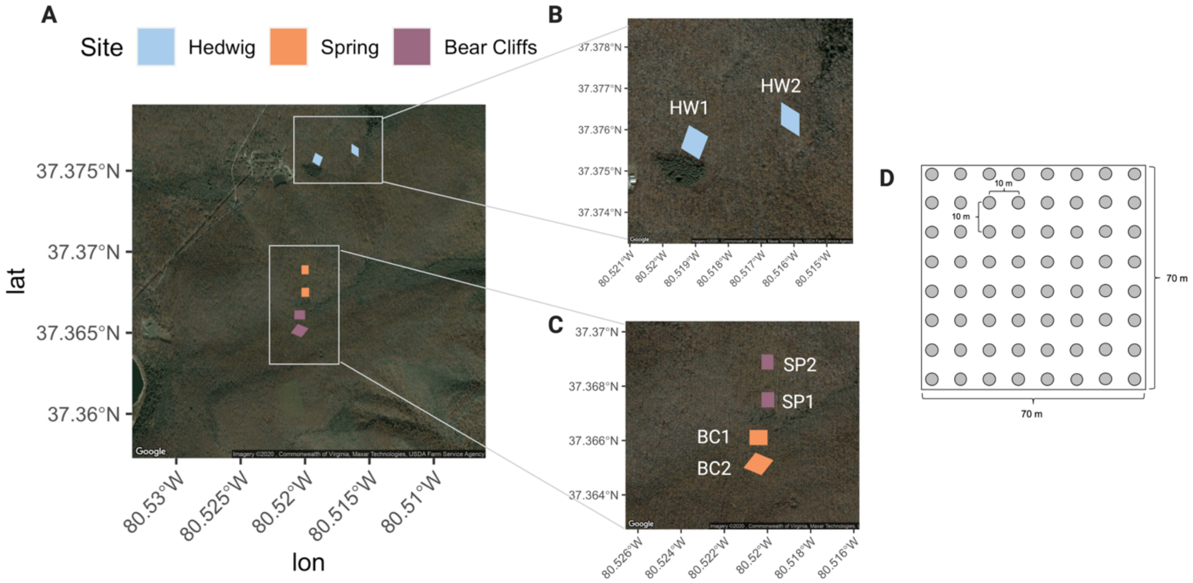

Figure S1. Grid layout for trapping sites included in field experiment analysis. A. Arrangement of all 3 trapping sites (6 grids) surround Mountain Lake Biological Station. B. Grid location of site Hedwig. C. Grid locations of sites Spring & Bear Cliffs. D. Schematic of trapping location layout within each grid.

In 2010, treatment was assigned at the population-level, where one of each pair of spatial replicate grids was assigned to anthelmintic treatment (all animals were treated) and the other assigned to control (5% sucrose solution). In 2011, treatment was assigned at the individual level for 4/6 grids and captured mice were assigned to one of four treatment groups: (i) ivermectin, (ii) fipronil, (iii) ivermectin + fipronil, or (iv) control. The remaining two grids were designated as control. Fipronil, a common insecticide that was administered topically, was used to reduce arthropod vectors (e.g. fleas) that can transmit additional microparasites as part of addition investigation into parasite community dynamics. However, we found no effect of fipronil treatment on either nematodes or SNV so these treatments were grouped as either received ivermectin (treated) or no treatment (control) for subsequent analyses.

Morphometric data collection was carried out as follows. Length (body, tail, hindfoot, ear) measurements were recorded, and *Peromyscus maniculatus* was distinguished from *P. leucopus* based on tail length exceeding body length, sharply bicoloured tail and a hair tuft at the end of the tail. Age (juvenile, sub-adult or adult) was assessed for both species by pelage color and molt patterns. Sex was determined by the urogenital distance. Mice were considered reproductively active if the females had a perforate vagina, were pregnant, or lactating; or testes were descended in males.

Retro-orbital bleeds were carried out under short-term anesthesia (Isoflurane, ~150 µl). Faecal samples were collected from individual traps at the end of each day, and traps were subsequently washed with hospital-grade detergent before re-use.

Laboratory assays

Fecal samples were weighed and stored in 10% formalin prior to parasite quantification. Blood samples were centrifuged to separate plasma and stored at -80°C until serology assays. SNV serology followed the standard CDC protocol, as follows. ELISA microtiter plates (Nunc Star Well Maxisorb, Thermo Scientific 441653) were coated overnight at 4°C with 100 µL per well of recombinant affinity purified SNV antigen (CDC Lot No. SPR569, 1:2000 dilution in phosphate buffered saline (PBS), 0.01 M, pH 7.4) on half of each plate and a control antigen (containing components of the cell lines used to produce the SNV antigen; CDC Lot No. SPR568, 1:2000 dilution in PBS) on the other half. Plates were then washed three times with PBS supplemented with 0.1% Tween-20 (PBST, pH 7.4). Serum samples were diluted 1:100 in ELISA diluent (PBST supplemented with 5% skim milk, pH 7.4) and added to wells. Positive control serum (anti-SNV NC antigen HMAF; CDC #703142) was used at an initial dilution of 1:1000,

and negative control serum (CDC # 703226) at 1:100, both in ELISA diluent. Positive and negative controls were included on each plate for each antigen.

After incubation of samples and controls at 37°C for 60 minutes, wells were washed three times with PBST. Peroxidase-labeled anti-*Rattus norvegicus* conjugate (Kirkegaard and Perry Laboratories, Cat No. 14-16-06, diluted 1:2500 in ELISA diluent) and peroxidase-labeled anti-*Peromyscus leucopus* IgG secondary antibody (Kirkegaard and Perry Laboratories, Cat No. 14-33-06, diluted in 1:2500 in ELISA diluent) were then added to all wells and incubated for 60 minutes at 37°C. Plates were washed three times and then ABTS peroxidase substrate (Kirkegaard and Perry Laboratories, Cat. Nos. 506400 & 506500, combined 1:1) was added (100 μL per well). Plates were then incubated for 30 minutes at 37°C. Spectrophotometric data were collected at 405nm on a Multiskan GO.

Serological testing was done in two stages. First, all samples were screened for seropositivity in quadruplicate wells. For each serum sample, we subtracted the mean OD values of the wells with control antigen from the mean OD values of the wells with the SNV antigen, to give a "net" positive adjusted OD value. Results were considered positive if this value was greater than 3 standard deviations above the adjusted OD405 (cut-off value) for the negative control samples.

Statistical analysis

We use a timepoint:treatment interaction here to test group differences. Individual ID was initially included in the treatment models as a random effect, but dropped given the very high percentage (>70%) of individuals which had only single captures, resulting in non-convergence of models estimating interindividual variation (Figure S2).

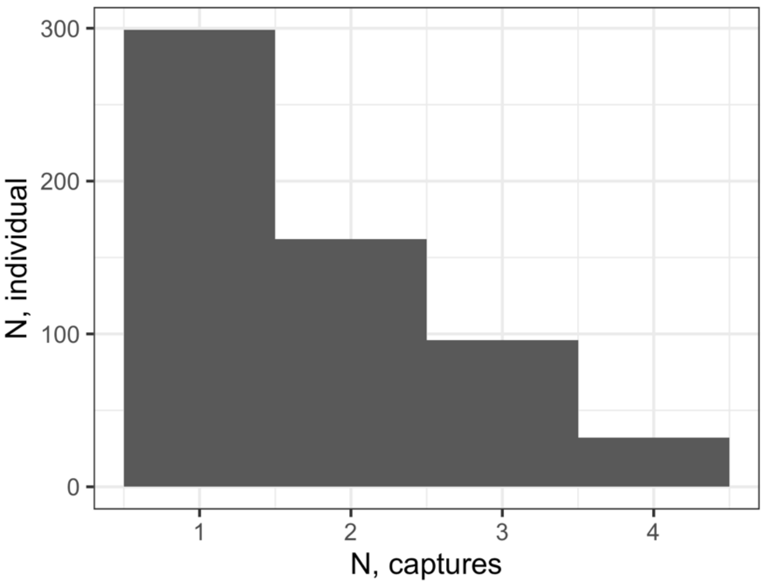

Figure S2. Histogram of capture frequency for mice included in field experiment. 299 (71.2%) mice were captured only once and 81 (19.3%) had two captures, with very few (32 and 8, respectively) having beyond 2 (3 or 4) captures.

Table S1. Model output for co-infection effects on host condition and survival. Blank cells indicate that a term was not included in the highlighted model.

	Condition		Survival	
	Weight, g		Recapture Duration	
variable	Estimate	p	Estimate	p
Trap Session			-0.04 (-0.14 - 0.06)	0.438
Weight, g			0.03 (0 - 0.06)	0.094
Reproductive, Yes	**1.62 (1.02 - 2.22)**	**<0.001**		
Ivermectin, Treated	-0.34 (-1.1 - 0.41)	0.375	0 (-0.31 - 0.32)	0.987
Year, 2011	-0.18 (-0.88 - 0.51)	0.6	-0.04 (-0.31 - 0.23)	0.767
Sex, Male	**-1.11 (-1.77 - -0.44)**	**0.001**	-0.1 (-0.35 - 0.15)	0.433
Age, Sub-adult	**-5.04 (-5.73 - -4.35)**	**<0.001**	0.2 (-0.11 - 0.51)	0.213
Species, *P. maniculatus*	-0.89 (-1.88 - 0.1)	0.079	-0.18 (-0.56 - 0.19)	0.335
Coinfection, Nem. only	0.66 (-0.1 - 1.41)	0.087	**0.34 (0.06 - 0.62)**	**0.016**
Coinfection, SNV only	1.28 (-0.07 - 2.63)	0.063	**0.74 (0.18 - 1.29)**	**0.009**
Coinfection, Co-infected	**3.37 (1.63 - 5.11)**	**<0.001**	**1.62 (1.04 - 2.19)**	**<0.001**
Intercept	**20.47 (19.51 - 21.43)**	**<0.001**	**2.87 (2.09 - 3.65)**	**<0.001**

Table S2. Goodness-of-fit for parasite and host fitness models.

Model Type	Model	Marginal R^2	Conditional R^2
Infection, First Capture	Nematode, probability	0.034	0.066
	Nematode, intensity	0.396	0.396
	SNV, probability	0.185	0.308
	SNV, antibody response	0.214	0.268
Infection, All Captures	Nematode, probability	0.115	0.139
	Nematode, intensity	0.350	0.438
	SNV, probability	0.179	0.319
	SNV, antibody response	0.207	0.285
Host Fitness	Condition	0.410	0.724
	Survival	0.289	0.390
